# Supplementary material for: Territorial bias in university rankings: a complex network approach
Source: Sci Rep. 2022 Mar 23;12:4995. doi: 10.1038/s41598-022-08859-w (PMC8943138; doi:10.1038/s41598-022-08859-w)
Supplement: Supplementary file 1 — Supplementary Information. [file 41598_2022_8859_MOESM1_ESM.pdf]

Supplementary Information

Territorial bias in university rankings: a complex network approach

Authors

Loredana Bellantuono,[1,2] Alfonso Monaco,[2] Nicola Amoroso,[3,2]* Vincenzo Aquaro,[4§] Marco Bardoscia,[5,6¶] Annamaria Demarinis Loiotile,[7,8] Angela Lombardi,[8,2] Sabina Tangaro,[9,2] Roberto Bellotti[8,2]

Affiliations

[1]Dipartimento di Scienze Mediche di Base, Neuroscienze e Organi di Senso, Università degli Studi di Bari Aldo Moro, 70124, Bari, Italy.
[2]Istituto Nazionale di Fisica Nucleare, Sezione di Bari, 70125, Bari, Italy.
[3]Dipartimento di Farmacia-Scienze del Farmaco, Università degli Studi di Bari Aldo Moro, 70125, Bari, Italy.
[4] Division for Public Institutions and Digital Government, United Nations Department of Economic and Social Affairs (DESA), New York, NY, 10017, USA.
[5]Bank of England, London EC2R 8AH, United Kingdom.
[6]Department of Computer Science, University College London, London WC1E 6BT, United Kingdom
[7]Dipartimento di Ingegneria Elettrica e dell'Informazione, Politecnico di Bari, 70125, Bari, Italy.
[8]Dipartimento Interateneo di Fisica, Università degli Studi di Bari Aldo Moro, 70126, Bari, Italy.
[9]Dipartimento di Scienze del Suolo, della Pianta e degli Alimenti, Università degli Studi di Bari Aldo Moro, 70126, Bari, Italy.

*Corresponding author. Email: nicola.amoroso@uniba.it

[§]The designations employed and the presentation of the material in this paper do not imply the expression of any opinion whatsoever on the part of the United Nations concerning the legal status of any country, territory, city or area, or of its authorities, or concerning the delimitation of its frontiers or boundaries. The designations "developed" and "developing" economics are intended for statistical convenience and do not necessarily imply a judgment about the state reached by a particular country or area in the development process. The term "country" as used in the text of this publication also refers, as appropriate, to territories or areas. The views expressed are those of the individual authors of the paper and do not imply any expression of opinion on the part of the United Nations.

[¶]Any views expressed are solely those of the author(s) and so cannot be taken to represent those of the Bank of England or to state Bank of England policy. This paper should therefore not be reported as representing the views of the Bank of England or members of the Monetary Policy Committee, Financial Policy Committee or Prudential Regulation Committee.

1 On bias detection and removal within OECD university networks

1.1 Assortativity scatter plots of OECD university networks

The scatter plots reported in Supplementary Fig. S1 provide a qualitative representation of the assortativity of the territorial network (left panels) and the educational offer network (right panels), with respect to the overall score and the specific dimensions of the THE rating. In each scatter plot, the point coordinates correspond to the THE scores of pairs of connected institutions, while the point color is determined by the strength of connections. Remarkably, the scatter plots concerning the territorial network, especially those related to the overall score, citations and international outlook dimensions, show a typical assortative pattern: most of the high-weight dots lie close to the main diagonal line, that represents connections between universities with the same THE score. On the other hand, in the scatter plots associated with the educational offer network, dots indicating strong connections are much more evenly distributed, for all the THE ratings. These results indicate that the territorial network shows a stronger tendency to exhibit connections between nodes with similar THE scores, compared to the educational offer one.

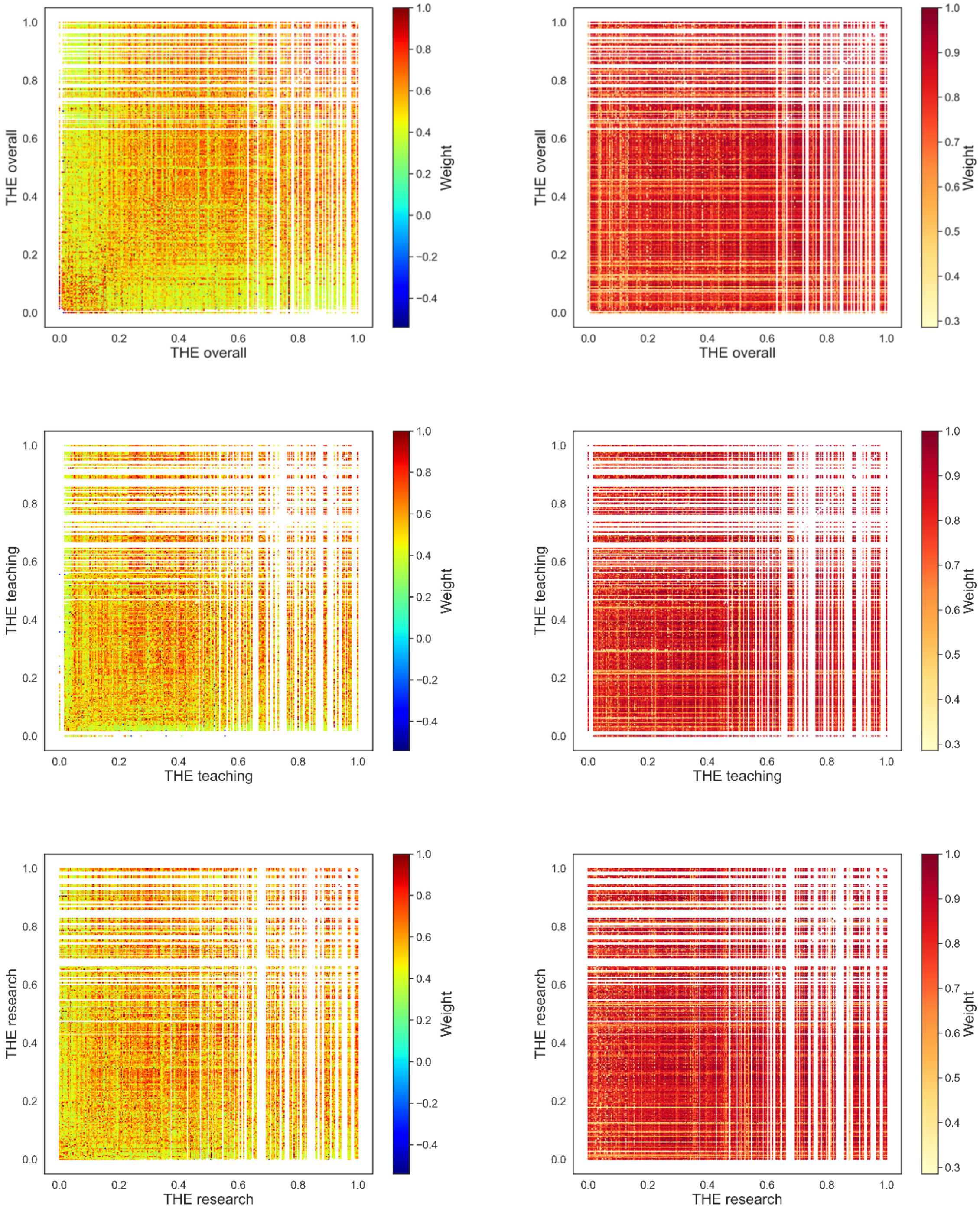
THE overall
THE overall
Weight
THE overall
THE overall
Weight
THE teaching
THE teaching
Weight
THE teaching
THE teaching
Weight
THE research
THE research
Weight
THE research
THE research
Weight

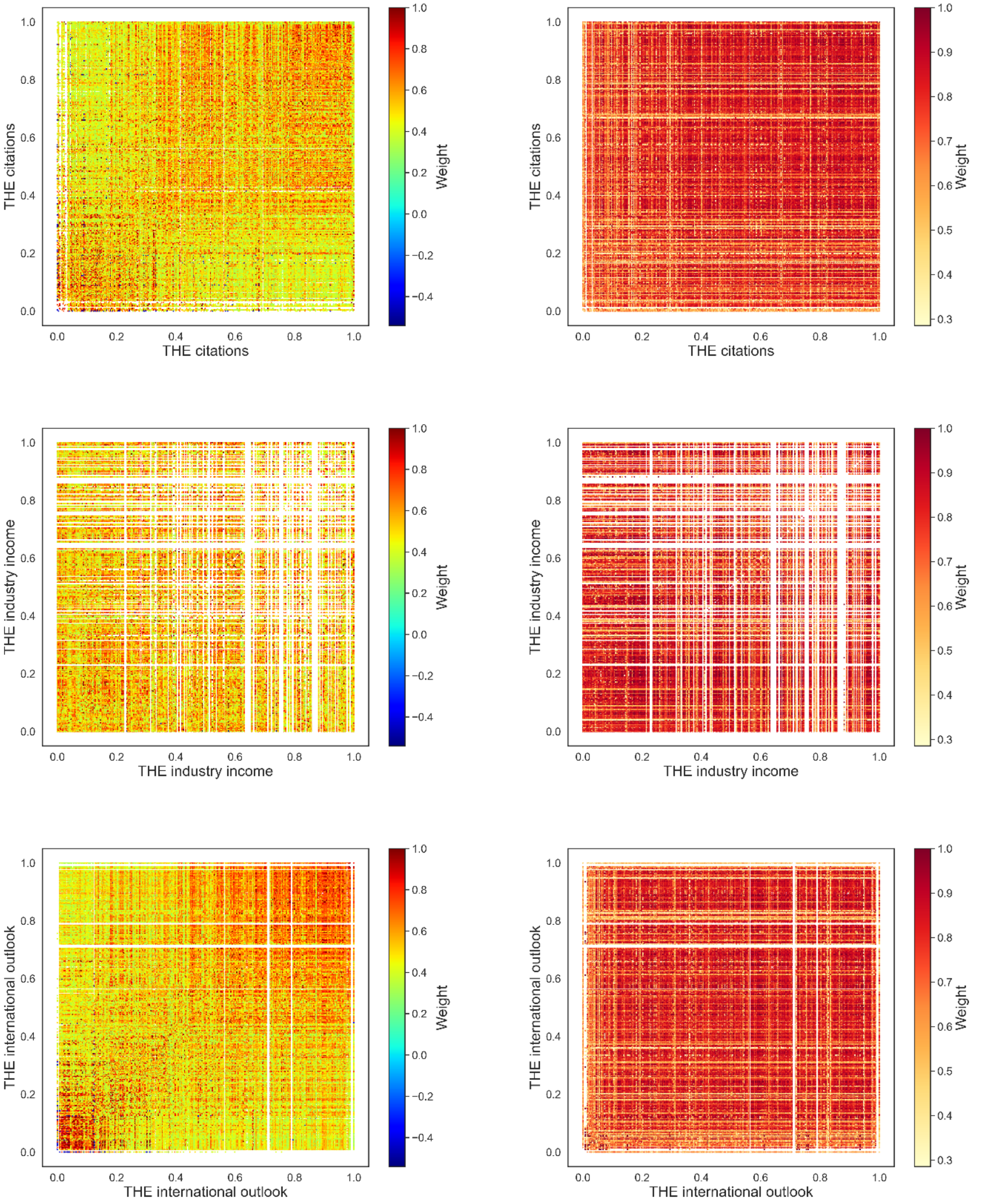

Fig. S1.
Assortativity scatter plots of OECD university networks with respect to the overall score and the specific dimensions of the THE rating. In these scatter plots each dot corresponds to one of the links in the territorial (left panels) and educational offer (right panels) OECD university network, and its coordinates along the horizontal and vertical axes represent the THE scores, for a specific ranking, of the two universities at either end of that link. A configuration in which high-weight links are mostly distributed close to the main diagonal, thus connecting universities with similar THE scores, reveals the existence of an assortative pattern, absent in the case of a more uniform distribution of weights.

1.2 Community detection workflow in OECD university networks

In this work, we perform community detection by a hierarchical algorithm, that starts finding a first-level partition of the network, and then proceeds to finding the partition of first-level communities, treated as sub-networks. The criteria to choose the optimal partition and the iteration level of the hierarchical process are discussed in the Materials and Methods section.

The first hierarchical level of subnational area community detection yields the partition

- OA1: 71 regions in Central and South America, United States, Turkey;
- OA2: 166 regions in Western Europe, North America, Oceania, Israel, Japan;
- OA3: 106 regions in Europe, Israel, Korea

as the most reliable result, obtained for $\gamma = 1$ with 89% agreement and $\langle NMI \rangle = 0.978$. At the second level of the hierarchy, we find

- OA1a: 38 regions in Argentina, Brazil, Colombia, Costa Rica, Mexico, Peru;
- OA1b: 33 regions in Chile, Mexico, Turkey, United States (south-east);
- OA2a: 105 regions in Australia, Western Europe, Israel, Japan, New Zealand;
- OA2b: 61 regions in Canada, United States, Australia, New Zealand, Japan, Estonia, Spain (Balearic Islands);
- OA3a: 57 regions in Eastern Europe, Korea;
- OA3b: 49 regions in Southern and Central Europe, Korea

resulting from the subdivision of OA1 in (OA1a,OA1b) with 96% agreement and $\langle NMI \rangle = 0.957$ for $\gamma = 0.9$, of OA2 in (OA2a,OA2b) with 95% agreement and $\langle NMI \rangle = 0.995$ for $\gamma = 0.95$, and of OA3 in (OA3a,OA3b) with 100% agreement for $\gamma = 0.9$. At the third level, the algorithm finds a partition in which some communities have a smaller than 5% share of the network, therefore the iteration stops at the second level. A geographical representation of the accepted partition is provided in Figure S2. To construct a partition in communities of the OECD universities, we associate each university with the region of its main seat, and collect all institutions related to geographical areas belonging to the same OA community. Therefore, we obtain the final subdivision, reported in the Results section of the main text:

- OT1a: 80 universities in Argentina, Brazil, Colombia, Costa Rica, Mexico, Peru;
- OT1b: 78 universities in Chile, Mexico, Turkey, United States (south-east);
- OT2a: 430 universities in Australia, Western Europe, Israel, Japan, New Zealand;
- OT2b: 241 universities in Canada, United States, Australia, New Zealand, Japan, Estonia, Spain (Balearic Islands);
- OT3a: 122 universities in Eastern Europe, Korea;
- OT3b: 137 universities in Southern and Central Europe, Korea.

Concerning the educational offer network, at the first level the network splits into three communities at $\gamma = 1$, with a rather reduced agreement (59%), but still with large mutual similarity between them ($\langle NMI \rangle = 0.957$); the resulting partition reads:

- OE1: 301 universities with an educational offer focused on engineering, economics and science;

- OE2: 427 universities with a very generalized range of educational offer;
- OE3: 360 universities with an educational offer characterized by underrepresented engineering areas;

The second-level partition is still acceptable: for $\gamma = 1$, OE1 splits in two communities (OE1a,OE1b) with 100% agreement, OE2 in two communities (OE2a,OE2b) with 96% agreement and $\langle NMI \rangle = 0.973$, and OA3 in three communities (OE3a,OE3b,OE3c) with 93% agreement and $\langle NMI \rangle = 0.947$. The final partition, reported in the Results section of the main text, reads:

- OE1a: 128 universities with predominant engineering and computer science areas, and underrepresented humanities, health and social science areas;
- OE1b: 173 universities with predominant science, engineering and economics areas;
- OE2a: 221 universities with a very generalized range of educational offer, not including veterinary science;
- OE2b: 206 universities with a very generalized range of educational offer, differing from OE2a due to the overrepresentation of "veterinary science" and "agriculture and forestry" areas, present in 100% and 92% of institutions, respectively;
- OE3a: 179 universities with a generalized range of educational offer, but underrepresented engineering areas;
- OE3b: 48 universities with an educational offer strongly focused on the health area;
- OE3c: 133 universities with predominant economics, humanities and social science areas.

The iteration stops at the second level also in this case, since at the third level the algorithm finds a partition in which some communities have a smaller than 5% share of the network. The full list of universities with their community membership in both the territorial and educational offer networks is reported in Supplementary Data S1.

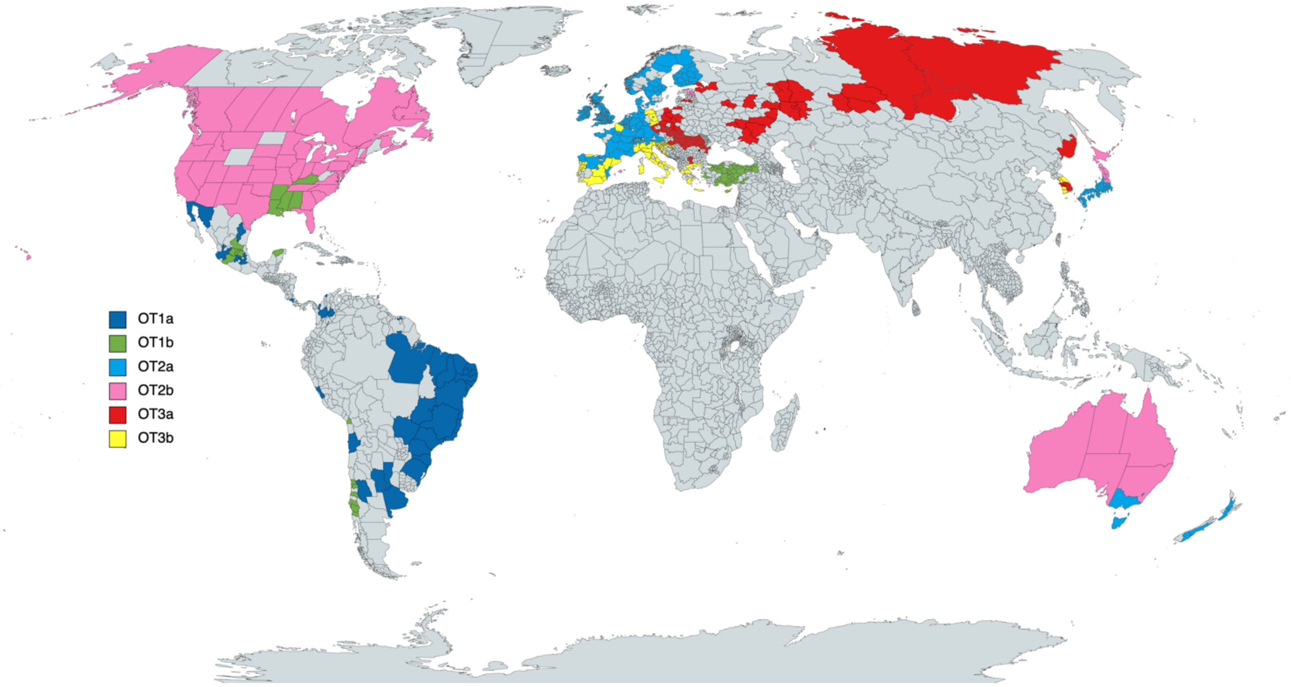

Fig. S2.

Map of communities in the OECD territorial area network. The OECD subregions involved in the analysis are colored according to their community membership. The map is generated with the MapChart online tool (67).

1.3 Debiasing parameters and principal component analysis for THE ranking dimensions

In Supplementary Fig. S3, we show the scatter plots in the planes (δ_T, δ_E) of the debiasing parameters referred to each THE ranking dimension. Points are colored according to their territorial community membership.

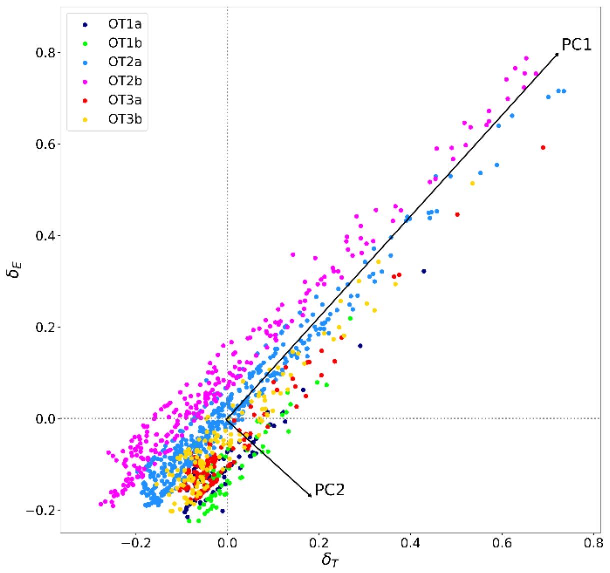

(a) THE teaching

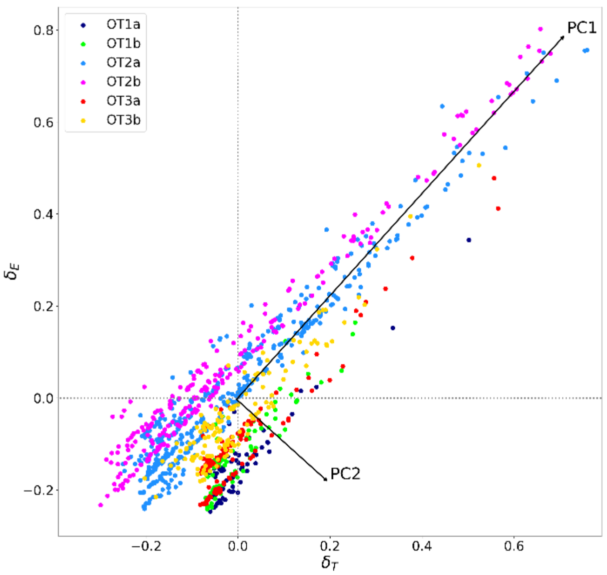

(b) THE research

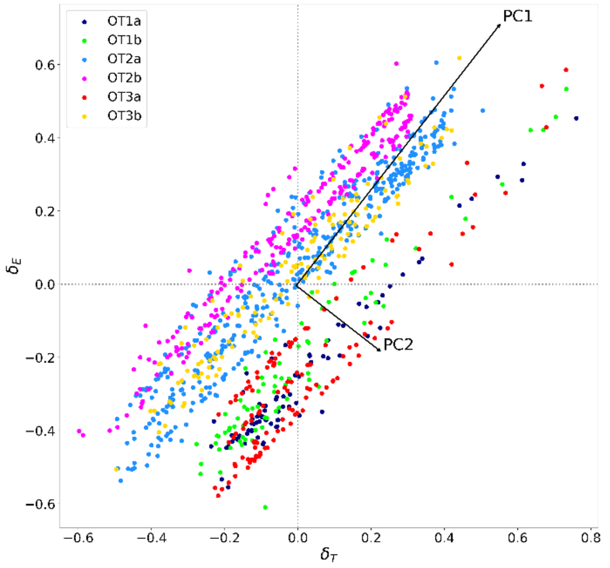

(b) THE citations

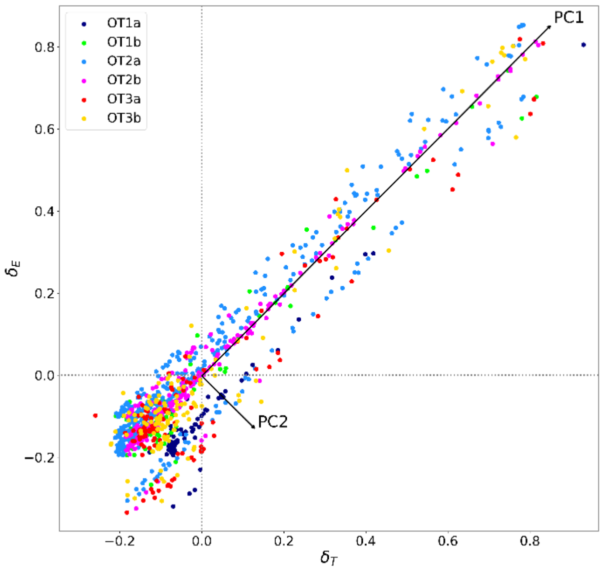

(d) THE industry income

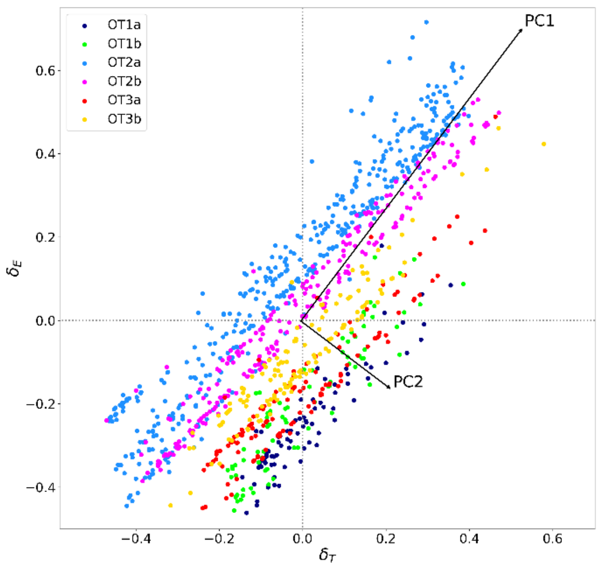

(e) THE international outlook

Fig. S3.

Territorial bias in the THE ranking dimensions. In all scatter plots, each dot corresponds to an OECD university, and its coordinates along the horizontal and vertical axes represent the debiasing parameters. The values δ_T and δ_E, referred to the specific dimension, assess the results achieved by the institution in the corresponding THE ranking, respectively by comparison with the rest of the OT and OE community it belongs to. Each dot in the scatter plot is colored according to its OT community membership.

We report in Table S1 the details of Principal Component Analysis (PCA) on the distribution of debiasing parameters associated to THE dimensions, namely the variance explained by each component and the Pearson correlation of each component with the GDP per capita PPP of the OECD subregion in which the main university seat is located. The related p-values are obtained by comparing the empirical Pearson correlation with the exact distribution of the correlation values between two random vectors, independent and normally distributed.

Table S1 also shows the assortativity values of territorial and educational offer network with respect to all the THE ranking dimensions, along with standard errors and p-values, computed according to the Student t-distribution hypothesis (see Materials and Methods for details).

The full list of OECD universities with the debiasing parameters (δ_T, δ_E) referred to each THE dimension, along with the related principal component values (PC1,PC2), is reported in Supplementary Data S2.

Table S1.

Properties of the principal components (PC1, PC2) of the $(\boldsymbol{\delta_T}, \boldsymbol{\delta_E})$ distributions associated to the THE rankings. Statistically significant values $(p < 10^{-2})$ of Pearson correlation and network assortativity are highlighted in boldface. Values of assortativity and Pearson correlation with GDP per capita PPP for the original rankings are reported for comparison.

	Explained variance (% of total)	Pearson correlation with GDP per capita PPP	Assortativity of territorial network	Assortativity of educational offer network
**THE overall score**				
**PC1**	91.4	$\mathbf{0.367}$ $(p < 10^{-9})$	$\mathbf{0.054 \pm 0.001}$ $(p < 10^{-9})$	0.001 ± 0.001 $(p = 0.455)$
**PC2**	8.6	$\mathbf{-0.523}$ $(p < 10^{-9})$	$\mathbf{0.227 \pm 0.001}$ $(p < 10^{-9})$	0.001 ± 0.001 $(p = 0.175)$
**Original ranking**		$\mathbf{0.481}$ $(p < 10^{-9})$	$\mathbf{0.109 \pm 0.001}$ $(p < 10^{-9})$	$\mathbf{0.003 \pm 0.001}$ $(p = 0.007)$
**THE teaching**				
**PC1**	94.8	$\mathbf{0.312}$ $(p < 10^{-9})$	$\mathbf{0.018 \pm 0.001}$ $(p < 10^{-9})$	0.001 ± 0.001 $(p = 0.265)$
**PC2**	5.2	$\mathbf{-0.572}$ $(p < 10^{-9})$	$\mathbf{0.203 \pm 0.001}$ $(p < 10^{-9})$	0.001 ± 0.001 $(p = 0.168)$
**Original ranking**		$\mathbf{0.413}$ $(p < 10^{-9})$	$\mathbf{0.043 \pm 0.001}$ $(p < 10^{-9})$	0.002 ± 0.001 $(p = 0.044)$
**THE research**				
**PC1**	94.1	$\mathbf{0.284}$ $(p < 10^{-9})$	$\mathbf{0.026 \pm 0.001}$ $(p < 10^{-9})$	0.001 ± 0.001 $(p = 0.354)$
**PC2**	5.9	$\mathbf{-0.568}$ $(p < 10^{-9})$	$\mathbf{0.219 \pm 0.001}$ $(p < 10^{-9})$	$\mathbf{0.004 \pm 0.001}$ $(p = 9 \cdot 10^{-5})$
**Original ranking**		$\mathbf{0.393}$ $(p < 10^{-9})$	$\mathbf{0.059 \pm 0.001}$ $(p < 10^{-9})$	0.002 ± 0.001 $(p = 0.015)$
**THE citations**				
**PC1**	90.5	$\mathbf{0.337}$ $(p < 10^{-9})$	$\mathbf{0.072 \pm 0.001}$ $(p < 10^{-9})$	0.001 ± 0.001 $(p = 0.580)$
**PC2**	9.5	$\mathbf{-0.476}$ $(p < 10^{-9})$	$\mathbf{0.204 \pm 0.001}$ $(p < 10^{-9})$	$\mathbf{0.004 \pm 0.001}$ $(p = 4 \cdot 10^{-5})$
**Original ranking**		$\mathbf{0.451}$ $(p < 10^{-9})$	$\mathbf{0.134 \pm 0.001}$ $(p < 10^{-9})$	$\mathbf{0.004 \pm 0.001}$ $(p = 10^{-4})$
**THE industry income**				
**PC1**	97.7	$\mathbf{0.106}$ $(p = 5 \cdot 10^{-4})$	$\mathbf{0.010 \pm 0.001}$ $(p < 10^{-9})$	0.001 ± 0.001 $(p = 0.597)$
**PC2**	2.3	$\mathbf{-0.280}$ $(p < 10^{-9})$	$\mathbf{0.066 \pm 0.001}$ $(p < 10^{-9})$	$\mathbf{0.044 \pm 0.001}$ $(p < 10^{-9})$
**Original ranking**		$\mathbf{0.127}$ $(p = 3 \cdot 10^{-5})$	$\mathbf{0.015 \pm 0.001}$ $(p < 10^{-9})$	$\mathbf{0.003 \pm 0.001}$ $(p = 0.003)$
**THE international outlook**				
**PC1**	91.4	$\mathbf{0.265}$ $(p < 10^{-9})$	$\mathbf{0.097 \pm 0.001}$ $(p < 10^{-9})$	0.000 ± 0.001 $(p = 0.944)$
**PC2**	8.6	$\mathbf{-0.399}$ $(p < 10^{-9})$	$\mathbf{0.205 \pm 0.001}$ $(p < 10^{-9})$	$\mathbf{0.005 \pm 0.001}$ $(p = 3 \cdot 10^{-6})$
**Original ranking**		$\mathbf{0.359}$ $(p < 10^{-9})$	$\mathbf{0.147 \pm 0.001}$ $(p < 10^{-9})$	0.002 ± 0.001 $(p = 0.038)$

2 On bias detection and removal within Italian university networks

2.1 Assortativity scatter plots of Italian university networks

The scatter plots shown in Supplementary Fig. S4 provide a qualitative representation of the assortativity of the territorial network (left panels) and the educational offer network (right panels), with respect to the overall score and the specific dimensions of the CENSIS rating. The distribution of points and colors in the scatter plots allows to investigate possible effects of the context (both geographical and educational) on the performance of universities in CENSIS rankings. Results are analogous to the ones already discussed for the OECD university environment: in the Italian case study, the territorial network tends to be more assortative than the educational offer one. In particular, pronounced assortativity patterns are observed in the territorial network with respect to the overall score and some specific dimensions, such as employability and international outlook, among the others.

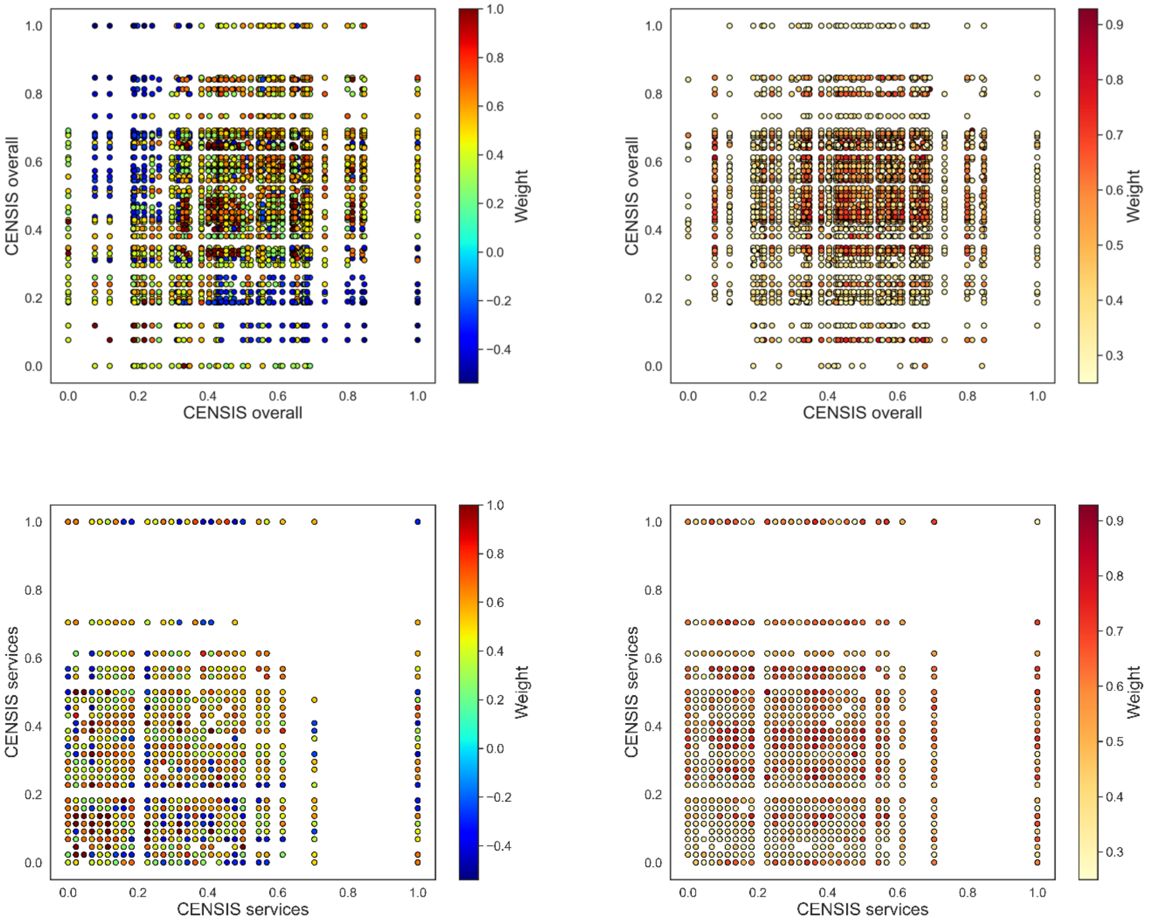

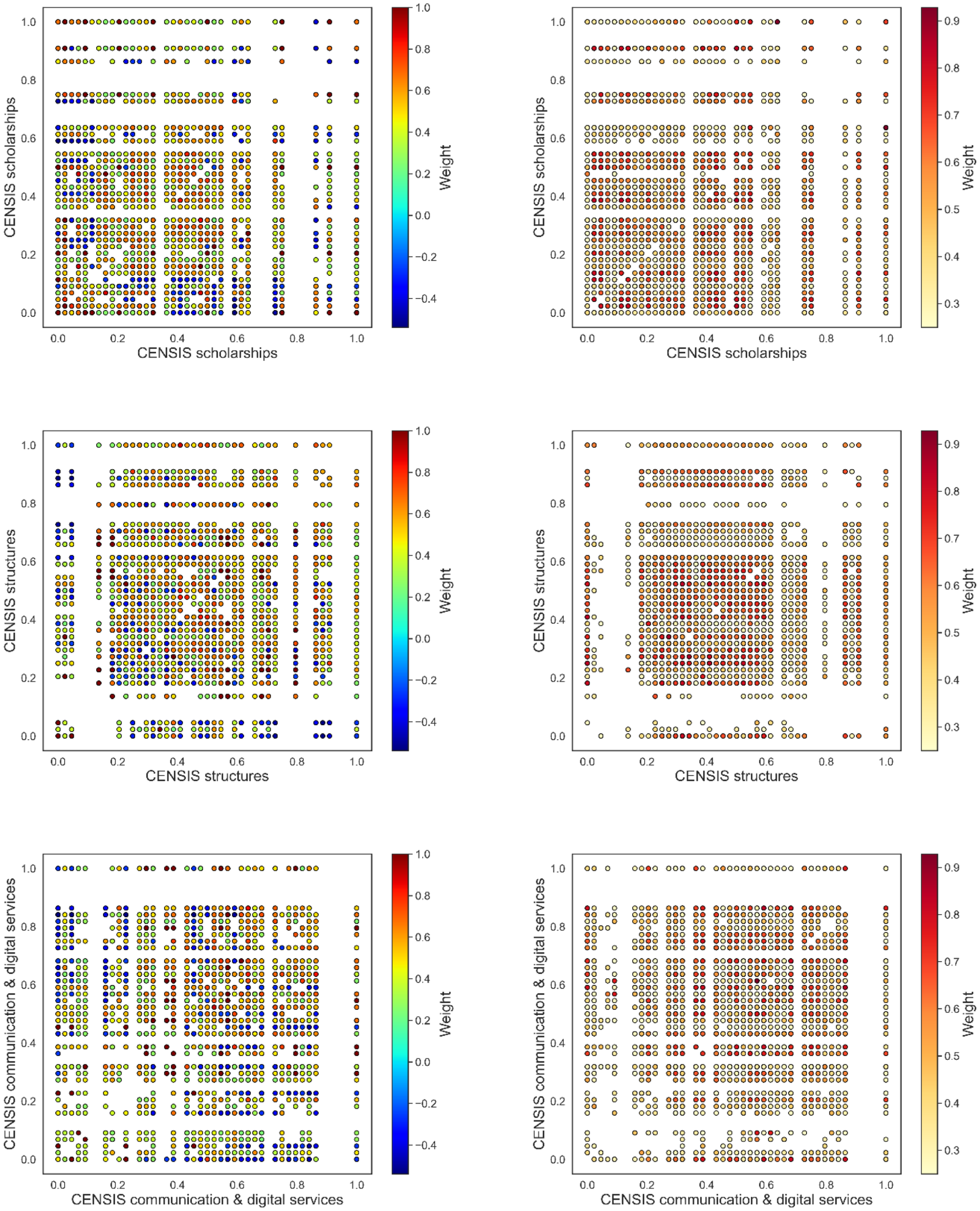
CENSIS scholarships
CENSIS scholarships
Weight
CENSIS structures
CENSIS structures
Weight
CENSIS communication & digital services
CENSIS communication & digital services
Weight

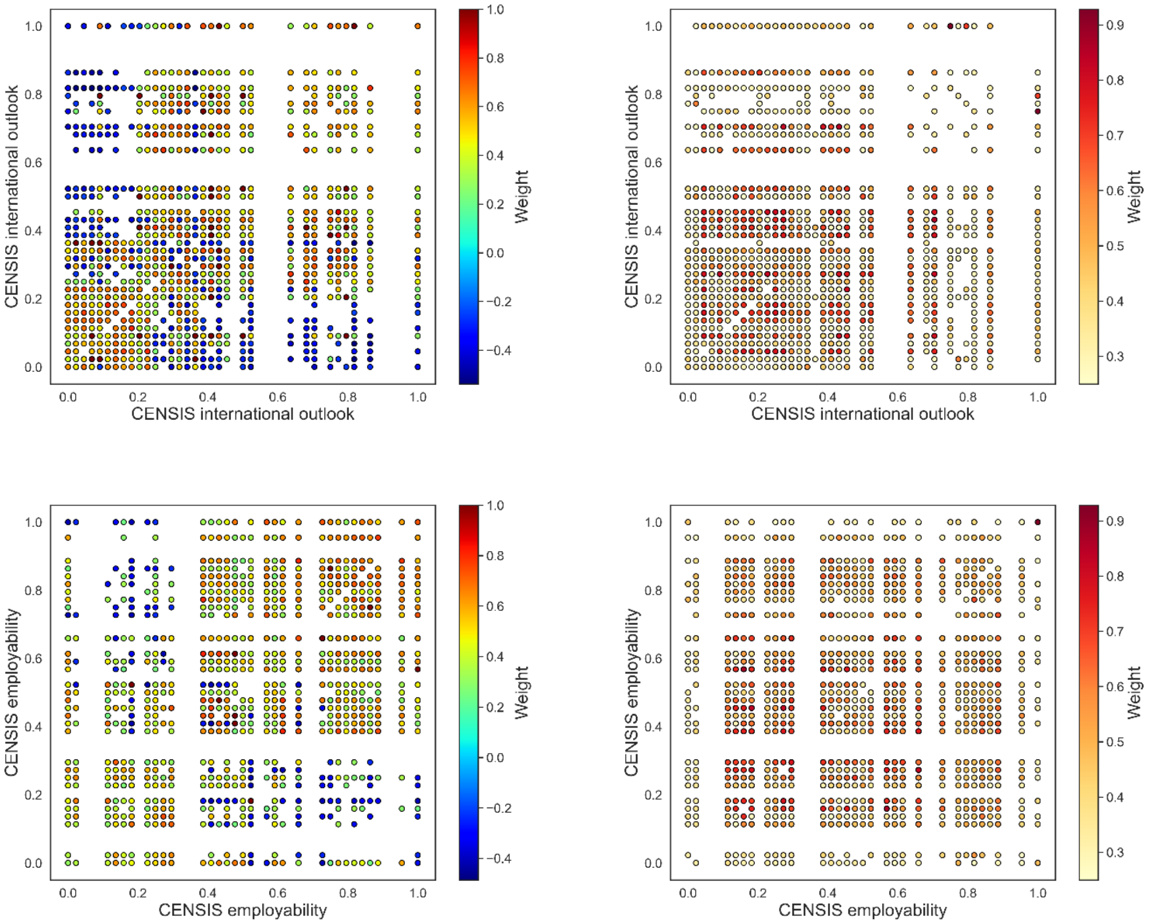

Fig. S4.

Assortativity scatter plots of Italian university networks with respect to the overall score and the specific dimensions of the CENSIS rating. In these scatter plots each dot corresponds to one of the links in the territorial (left panels) and educational offer (right panels) Italian university network, and its coordinates along the horizontal and vertical axes represent the CENSIS scores, for a specific ranking, of the two universities at either end of that link. A configuration in which high-weight links are mostly distributed close to the main diagonal, thus connecting universities with similar CENSIS scores, reveals the existence of an assortative pattern, absent in the case of a more uniform distribution of weights.

2.2 Community detection workflow in Italian university networks

Hierarchical community detection is performed for the Italian university networks, in the same spirit and with the same procedure as in the OECD case. The first hierarchical level of community detection on the subnational area network provides the following partition:

- IA1: 30 provinces in the center-north of the country, including Rome;
- IA2: 23 provinces in the center-south of the country,

with unanimous agreement (therefore, $\langle NMI \rangle = 1$), for all resolutions γ in [0.8,1] Such a partition reflects once more the historical and economic gap between North and South of Italy. At the second level, we find another partition with a dominant interpretation in terms of size and geographical location:

- IA1a: 21 provinces in the center-north with a small administrative center;
- IA1b: 9 provinces in the center-north, mostly with a large or historically relevant administrative center;
- IA2a: 10 provinces in center-south and Sardinia;
- IA2b: 13 provinces in the south.

The second-level partitions of IA1 in (IA1a,IA1b) and of IA2 in (IA2a,IA2b) are reached with 100% agreement at $\gamma = 0.95$ and $\gamma = 1$, respectively. The iteration stops at this stage since the partitions with largest consensus of second-level communities, returned at the next level, are trivial. A geographical representation of the accepted partition is provided in Figure S5. As in the OECD case, we associate each university with the province of its main seat, and collect all institutions related to provinces belonging to the same subnational area community. Therefore, we obtain the final subdivision, reported in the Results section of the main text:

- IT1a: 25 universities in center-north provinces with a small administrative center;
- IT1b: 35 universities in center-north provinces, mostly with a large or historically relevant administrative center;
- IT2a: 13 universities in center-south and Sardinia;
- IT2b: 19 universities in the south.

As concerns the educational offer network, the approach here adopted for community detection returns the following subdivision, reported in the Results section of the main text:

- IE1: 31 small, telematic universities, oriented to law, economics or foreign languages;
- IE2: 44 medium-to-large general-purpose universities;
- IE3: 9 polytechnic and small engineering-oriented universities;
- IE4: 8 research hospitals and health-oriented small universities,

with unanimous agreement (hence, $\langle NMI \rangle = 1$) at $\gamma = 1$. The partition at the following level is rejected, as it contains communities with less than 5% of nodes of the whole network. The full list of universities with their community membership in both the territorial and educational offer networks is reported in Supplementary Data S3.

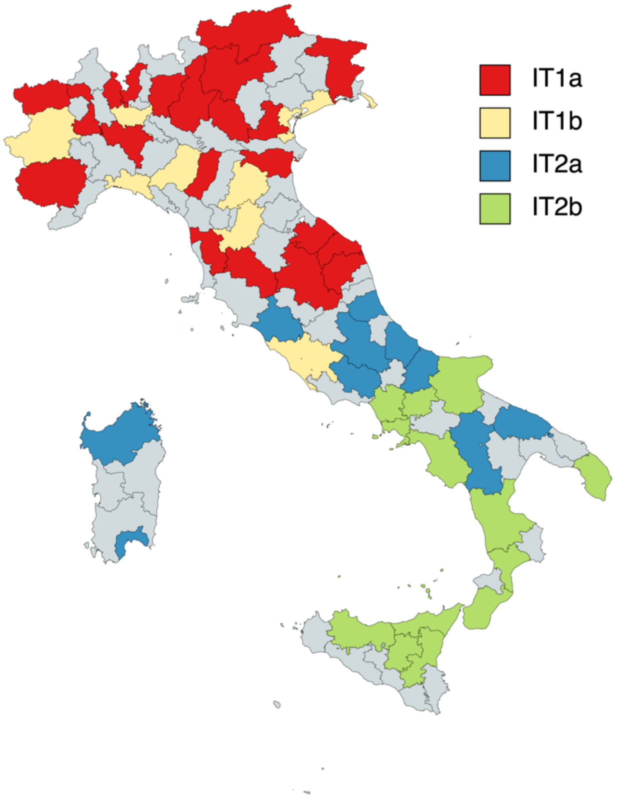

Fig. S5.

Map of communities in the Italian territorial area network. The Italian provinces are colored according to their community membership. The map is generated with the MapChart online tool (67).

2.3 Debiasing parameters and principal component analysis for CENSIS ranking dimensions

In Supplementary Fig. S6, we show the scatter plots in the planes (δ_T, δ_E) of the debiasing parameters referred to each CENSIS ranking dimension. Points are colored according to their territorial community membership.

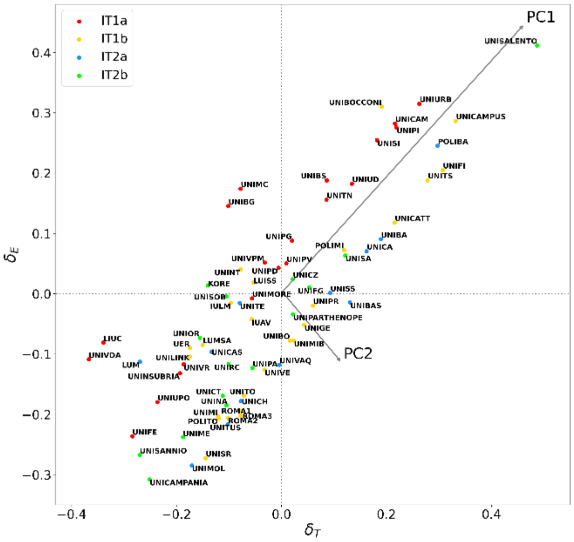

(a) CENSIS services

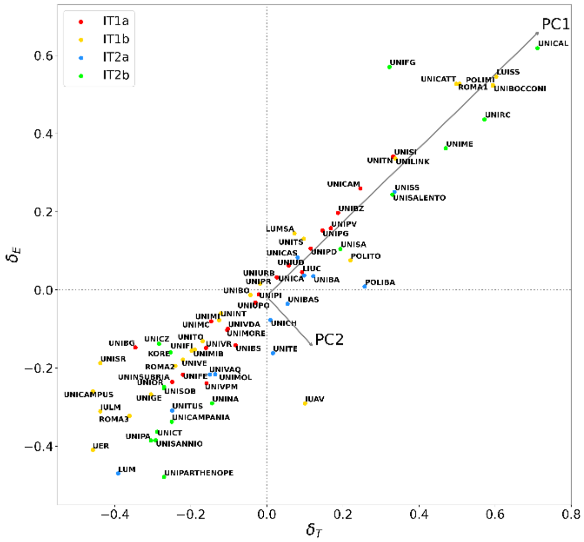

(b) CENSIS scholarships

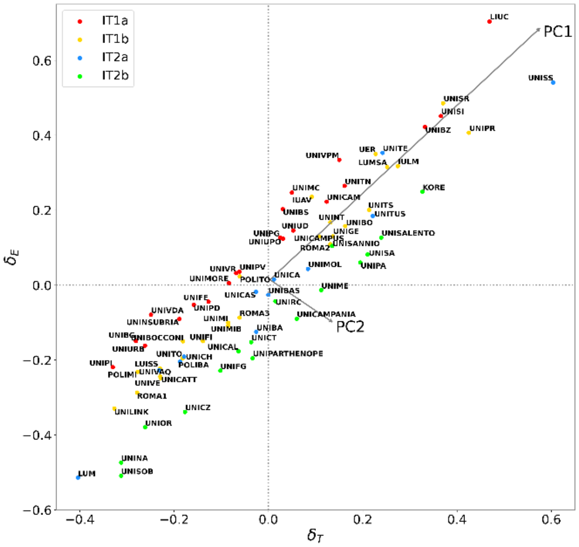

(c) CENSIS structures

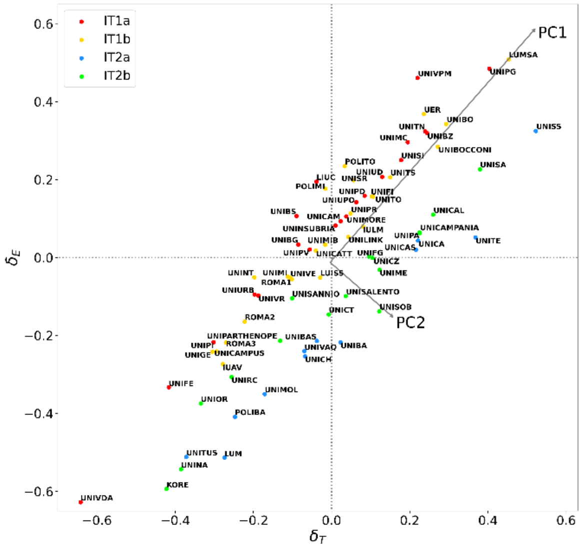

(d) CENSIS communication & digital services

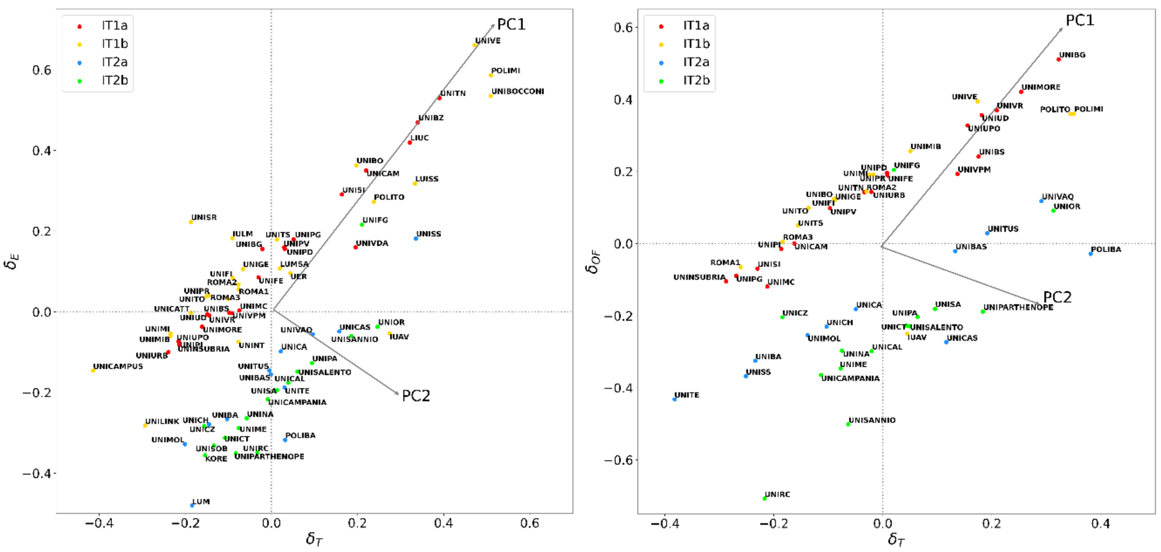

(e) CENSIS international outlook

(f) CENSIS employability

Fig. S6.

Territorial bias in the CENSIS ranking dimensions. In all scatter plots, each dot corresponds to an Italian university, and its coordinates along the horizontal and vertical axes represent the debiasing parameters. The values δ_T and δ_E, referred to the specific dimension, assess the results achieved by the institution in the corresponding CENSIS ranking, respectively by comparison with the rest of the IT and IE community it belongs to. Each dot in the scatter plot is colored according to its IT community membership.

We report in Table S2 the details of PCA on the distribution of debiasing parameters associated to CENSIS dimensions, namely the variance explained by each component and the Pearson correlation of each component with the average per capita available income of the province in which the main university seat is located. The related p-values are obtained by comparing the empirical Pearson correlation with the exact distribution of the correlation values between two random vectors, independent and normally distributed.

Table S2 also shows the assortativity values of territorial and educational offer network with respect to all the CENSIS ranking dimensions, along with standard errors and p-values, computed according to the Student t-distribution hypothesis (see Materials and Methods for details).

The full list of Italian universities with the debiasing parameters (δ_T, δ_E) referred to each CENSIS dimension, along with the related principal component values (PC1,PC2), is reported in Supplementary Data S4.

Table S2.

Properties of the principal components (PC1, PC2) of the $(\boldsymbol{\delta_T}, \boldsymbol{\delta_E})$ distributions associated to the CENSIS rankings. Statistically significant values $(p < 10^{-2})$ of Pearson correlation and network assortativity are highlighted in boldface. Values of assortativity and Pearson correlation with per capita available income for the original rankings are reported for comparison.

	Explained variance (% of total)	Pearson correlation with per capita available income	Assortativity of territorial network	Assortativity of educational offer network
**CENSIS overall score**				
**PC1**	87.2	$\mathbf{0.405}$ $(p = 3 \cdot 10^{-4})$	$\mathbf{0.113 \pm 0.019}$ $(p = 4 \cdot 10^{-9})$	-0.027 ± 0.017 $(p = 0.110)$
**PC2**	12.8	$\mathbf{-0.636}$ $(p = 10^{-9})$	$\mathbf{0.450 \pm 0.017}$ $(p < 10^{-9})$	0.005 ± 0.017 $(p = 0.785)$
**Original ranking**		$\mathbf{0.553}$ $(p = 3 \cdot 10^{-7})$	$\mathbf{0.289 \pm 0.018}$ $(p < 10^{-9})$	-0.019 ± 0.017 $(p = 0.275)$
**CENSIS services**				
**PC1**	94.0	0.066 $(p = 0.575)$	-0.017 ± 0.019 $(p = 0.387)$	-0.014 ± 0.017 $(p = 0.419)$
**PC2**	6.0	-0.051 $(p = 0.501)$	$\mathbf{0.064 \pm 0.019}$ $(p = 9 \cdot 10^{-4})$	$\mathbf{0.078 \pm 0.017}$ $(p = 6 \cdot 10^{-6})$
**Original ranking**		0.072 $(p = 0.544)$	$\mathbf{0.289 \pm 0.018}$ $(p < 10^{-9})$	-0.019 ± 0.017 $(p = 0.275)$
**CENSIS scholarships**				
**PC1**	96.9	0.079 $(p = 0.501)$	-0.036 ± 0.019 $(p = 0.064)$	-0.019 ± 0.017 $(p = 0.271)$
**PC2**	3.1	$\mathbf{-0.372}$ $(p = 10^{-3})$	$\mathbf{0.082 \pm 0.019}$ $(p = 2 \cdot 10^{-5})$	0.029 ± 0.017 $(p = 0.090)$
**Original ranking**		0.186 $(p = 0.113)$	0.027 ± 0.019 $(p = 0.161)$	-0.001 ± 0.017 $(p = 0.954)$
**CENSIS structures**				
**PC1**	96.2	0.145 $(p = 0.217)$	0.015 ± 0.019 $(p = 0.430)$	-0.025 ± 0.017 $(p = 0.151)$
**PC2**	3.8	$\mathbf{-0.530}$ $(p = 10^{-6})$	$\mathbf{0.481 \pm 0.017}$ $(p < 10^{-9})$	-0.014 ± 0.017 $(p = 0.423)$
**Original ranking**		0.217 $(p = 0.064)$	$\mathbf{0.077 \pm 0.019}$ $(p = 6 \cdot 10^{-5})$	-0.020 ± 0.017 $(p = 0.243)$
**CENSIS communication & digital services**				
**PC1**	93.1	0.244 $(p = 0.037)$	0.016 ± 0.019 $(p = 0.404)$	-0.022 ± 0.017 $(p = 0.197)$
**PC2**	6.9	$\mathbf{-0.670}$ $(p < 10^{-9})$	$\mathbf{0.501 \pm 0.017}$ $(p < 10^{-9})$	-0.013 ± 0.017 $(p = 0.460)$
**Original ranking**		$\mathbf{0.405}$ $(p = 3 \cdot 10^{-4})$	$\mathbf{0.122 \pm 0.019}$ $(p < 10^{-9})$	-0.012 ± 0.017 $(p = 0.475)$
**CENSIS international outlook**				
**PC1**	85.5	$\mathbf{0.432}$ $(p = 10^{-4})$	$\mathbf{0.118 \pm 0.019}$ $(p < 10^{-9})$	-0.006 ± 0.019 $(p = 0.757)$
**PC2**	14.5	$\mathbf{-0.705}$ $(p < 10^{-9})$	$\mathbf{0.412 \pm 0.018}$ $(p < 10^{-9})$	0.017 ± 0.019 $(p = 0.365)$
**Original ranking**		$\mathbf{0.591}$ $(p = 3 \cdot 10^{-8})$	$\mathbf{0.288 \pm 0.018}$ $(p < 10^{-9})$	0.007 ± 0.017 $(p = 0.667)$
**CENSIS employability**				
**PC1**	81.3	$\mathbf{0.561}$ $(p = 5 \cdot 10^{-6})$	$\mathbf{0.231 \pm 0.023}$ $(p < 10^{-9})$	-0.014 ± 0.019 $(p = 0.450)$
**PC2**	18.7	$\mathbf{-0.552}$ $(p = 7 \cdot 10^{-6})$	$\mathbf{0.318 \pm 0.023}$ $(p < 10^{-9})$	0.044 ± 0.019 $(p = 0.023)$
**Original ranking**		$\mathbf{0.715}$ $(p < 10^{-9})$	$\mathbf{0.436 \pm 0.022}$ $(p < 10^{-9})$	-0.003 ± 0.019 $(p = 0.865)$

2.4 Application of Gaussian Mixture model to the principal components of the debiasing parameters associated to the CENSIS overall score

To investigate the possible multimodal nature of the principal components of the debiasing parameters associated to the CENSIS overall score, we use a family of unidimensional Gaussian Mixture models, characterized by a number n_c of components that varies from 1 to 10. We report here for completeness the detailed outcomes of the Shapiro-Wilk test (87) and of the AIC and BIC score minimization (88), which is applied if the Shapiro-Wilk test is passed for more than one n_c:

- For PC1, all the components properly pass the Shapiro-Wilk test only in the case $n_c = 1$. For $n_c = 6,7,8,9$, some components pass the test, while it cannot be performed on other components, since they have less than 4 elements.
- For PC2, all the components properly pass the test for all the n_c's from 1 to 5, and from 7 to 10, while the case $n_c = 6$ involves components with less than 4 elements. The models that minimize the AIC and BIC scores are those with $n_c = 1$ and $n_c = 2$, respectively. Specifically, $n_c = 1$ minimizes the BIC (-137.26, against -131.06 of $n_c = 2$), while $n_c = 2$ minimizes the AIC (-142.58, against -141.87 of $n_c = 1$). In any case, the result for $n_c = 2$ allows to estimate the bias, evaluated as the horizontal distance 0.147 between the peaks of the two Gaussians.

3 Datasets used to generate university networks

3.1 Data collection for the OECD academic system

In this section, we provide information on data used to construct the OECD university networks. In Table S3, we introduce a list of the territorial indicators, compiled by the Organisation for Economic Co-operation and Development (OECD), used for the construction of the international territorial area network. Table S4 reports a list of the categories used by Times Higher Education to compile their rankings by educational area: this classification provides the basis to construct the educational offer network.

Table S3.

Territorial indicators, compiled by the Organisation for Economic Co-operation and Development (OECD), used for the construction of the international territorial area network. The dataset contains a total of 103 indicators referred to each OECD subregion (75). Indicators that do not pass the redundancy selection process are highlighted in light red. For each indicator and subregion, the OECD Regional Statistics database provides the most recent value available.

**Indicator**
3-year survival rate of firms (%)
Active physicians rate (active physicians per 1 000 people)
Annual growth rate of real GDP per capita (%, from to 2008 to 2016-17)
Annual growth rate of real GVA per worker (%, from to 2008 to 2016-17)
Artificial areas as a percentage of total area in 2015
Average disposable income per day of the first quintile (equivalised household, in USD PPP, constant prices of 2010)
Average disposable income per equivalised household (in USD PPP, constant prices of 2010)
Change in cooling degree-days needed to maintain an average building indoor temperature of 22 degree Celsius, from 1970-1984 to 2004-2018
Change in cropland (from 1992 to 2015, percentage points)
Change in heating degree-days needed to maintain an average building indoor temperature of 15.5 degree Celsius, from 1970-1984 to 2004-2018
Change in tree cover (from 1992 to 2015, percentage points)
Change in water bodies (from 1992 to 2015, percentage points)
CO2 emissions per electricity production (in tons of CO2 equivalent per gigawatt hours)
Coastal area as a percentage of total area
Cooling degree-days needed to maintain an average building indoor temperature of 22 degree Celsius, 2004-2018
Cropland as a percentage of total area in 2015
Decrease in Gini index due to transfers and taxes (%)
Decrease in poverty rates (national poverty line) due to transfers and taxes (%)
Decrease in poverty rates (regional poverty line) due to transfers and taxes (%)
Difference between built-up area growth rate and population growth rate (percentage points)
Employment in high-technology manufacturing as a percentage of total manufacturing employment
Employment in knowledge-intensive services as a percentage of total employment
Employment rate associated to newly created firms (%)

Employment rate of the foreign-born (%)
Exposure to PM2.5 in µg/m³, population weighted (micrograms per cubic metre)
Female research and development personnel as a percentage of total research and development employment
Firm creation rate (%)
Gender gap in employment rate (male-female, percentage points)
Gender gap in employment rate for the foreign-born (male-female, percentage points)
Gender gap in part-time employment incidence (female-male, percentage points)
Gender gap in the rate of early leavers (male-female, percentage points)
Gender gap in the rate of young population (from 18 to 24 years old) not in education, employment or training (NEET) (female-male, percentage points)
Gender gap in unemployment rate (female-male, percentage points)
Gini index of disposable income (after taxes and transfers) (from 0 to 1)
Gross Value Added (GVA) in Manufacture (ISIC rev4) as a percentage of GDP
Growth in disposable income per capita (%, from 2008 to 2016-17)
Heating degree-days needed to maintain an average building indoor temperature of 15.5 degree Celsius, 2004-2018
Homicides per 100 000 persons
Hospital beds rate (hospital beds per 10 000 people)
Increase in artificial areas (from 1992 to 2015, percentage points)
Infant mortality rate (number of deaths of children one year old or younger per 1 000 live births)
Life expectancy at birth (years)
Long term unemployment incidence (%)
Manufacturing employment as a percentage of total employment
Median disposable income per equivalised household (in USD PPP, constant prices of 2010)
Mortality rates for the 0 to 4 years old population (deaths per 10 000 people)
Municipal waste rate (kilos per capita)
Net firm creation rate (%) (firm birth rate minus firm death rate)
Number of motor road vehicles per 100 people
Over-qualification rates for the foreign-born (%)
Part-time employment incidence (%)
Patent applications (PCT) per 1 000 000 people
Percentage of early leavers from education and training, for the 18 to 24 years old population
Percentage of foreign-born among the total population
Percentage of household expenses dedicated to housing costs
Percentage of households with broadband internet access
Percentage of labour force with at least secondary education
Percentage of labour force with at least tertiary education
Percentage of people exposed to more than 10 µg/m³ (micrograms per cubic metre) of PM2.5
Percentage of people satisfied with the availability or quality of healthcare
Percentage of population from 15 to 19 years old enrolled in public or private institutions
Percentage of population from 25 to 64 years old participating in education and training
Percentage of population from 25 to 64 years old with at least tertiary education
Percentage of population satisfied with affordability of housing
Percentage of population satisfied with efforts to deal with poverty

Percentage of population satisfied with efforts to preserve the environment
Percentage of population satisfied with quality of air
Percentage of population satisfied with quality of water
Percentage of population satisfied with roads and highways
Percentage of population satisfied with the quality of public transportation systems
Percentage of population that believe corruption is spread throughout the government in the country
Percentage of population that believe women are treated with respect and dignity in their country
Percentage of population that believes their place of residence is a good place to live for gay or lesbian people
Percentage of population that believes their place of residence is a good place to live for migrants
Percentage of population that believes their place of residence is a good place to live for racial and ethnic minorities
Percentage of population that feel safe walking alone at night around the area they live
Percentage of population that have been assaulted or mugged in the previous 12 months
Percentage of population that have confidence in judicial system and courts
Percentage of population that have confidence in the local police force
Percentage of population that have confidence in the national government
Percentage of population with a disposable income below the 60% of national median disposable income
Percentage of population with a disposable income below the 60% of regional median disposable income
Percentage of total electricity production that comes from coal
Percentage of total electricity production that comes from fossil fuels (natural gas and oil, excluding coal)
Percentage of total electricity production that comes from nuclear power
Percentage of total electricity production that comes from renewable sources
Percentage of young population (from 18 to 24 years old) not in education, employment or training (NEET)
Productivity (Gross Value Added per worker) in agriculture, forestry and fishing (ISIC rev4) (in constant 2010 USD PPP)
Productivity (Gross Value Added per worker) in Manufacture (ISIC rev4) (in constant 2010 USD PPP)
Protected coastal area as a percentage of total coastal area
Ratio between average disposable income of top and bottom quintiles
Research and Development expenditure as a proportion of GDP (%)
Research and development personnel as a share of total employment
Rooms per person
Satisfaction with life as a whole (from 0 to 10)
Share of PCT co-patent applications that are done with foreign regions (in % of co-patent applications)
Terrestrial protected areas as a percentage of total area
Transport-related mortality rates (deaths per 100 000 people)
Tree cover as a percentage of total area in 2015

Unemployment rate (%)
Unemployment rate of the foreign-born (%)
Water bodies as percentage of total area in 2015
Youth unemployment rate (%)

Table S4.

Educational offer categories for the OECD case. The table reports a list of the categories used by Times Higher Education to compile their rankings by educational area (65).

THE educational offer sector
Accounting and finance
Agriculture and forestry
Archaeology
Architecture
Art performing, arts and design
Biological sciences
Business and management
Chemical engineering
Chemistry
Civil engineering
Communication and media studies
Computer science
Economics and econometrics
Education
Electrical and electronic engineering
General engineering
Geography
Geology, environmental, Earth and marine sciences
History, philosophy and linguistics
Law
Mathematics and statistics
Mechanical and aerospace engineering
Medicine and dentistry
Other health
Physics and astronomy
Politics and international studies (incl. development studies)
Psychology
Sociology
Sport science
Veterinary science

3.2 Data collection focusing on the Italian academic system

In this section, we provide information on data used to construct the Italian university networks. In Table S5, we introduce a list of the territorial indicators, compiled by the Italian National Institute of Statistics (ISTAT), on which the territorial area network is based. Each indicator is reported along with the reference year from which its value is retrieved. In Table S6, we report the classification of the Italian degree categories (*classi di laurea*) in terms of "broad fields" of the International Standard Classification of Education (ICSED).

Table S5.

Territorial indicators, compiled by the Italian National Institute of Statistics (ISTAT), used for the construction of the Italian territorial area network, and their reference year. The dataset contains a total of 144 indicators referred to each Italian province (78,79). For each indicator, data from the most recent year available are considered, with possible integrations of missing entries borrowed from at most 5 years before. Indicators that do not pass the redundancy selection process are highlighted in light red; the indicator "Average per capita income (EUR)", that is removed to constitute a benchmark for the wealth of provinces, is highlighted in light blue.

**Indicator**	**Year**
Territorial indicators for development policies	
Length of coasts not accessible for bathing (% of total length of coasts)	2008
Forest area (ha)	2005
Burnt forest area (% of total area)	2010
Population at risk from landslides (inhabitants per m^2)	2017
Population at risk from flooding (inhabitants per m^2)	2017
Visitors of national institutes of antiquities and art (average per institute)	2018
Visitors of national institutes of antiquities and art (per km^2)	2018
Paying/non-paying visitor ratio of national institutes of antiquities and art	2018
Sold tickets for theatre and musical activities (per 100 inhabitants)	2007
Visitors of national institutes of antiquities and art belonging to circuits (% of total)	2018
Tickets for museums belonging to circuits (% of total)	2018
Visitors of non-state institutes of antiquities and art (thousands)	2017
Visitors of non-state institutes of antiquities and art (per km^2)	2017
Employees of cooperative societies (% of total employees)	2016
Air quality monitoring stations (per 100,000 inhabitants)	2012
Petty-crime related offenses in cities (per 1,000 inhabitants)	2016
Petty-crime related offenses in cities, regional admin. centers (per 1,000 inhabitants)	2016
Petty-crime related offenses in cities (% of offenses)	2016
Petty-crime related offenses in cities, regional admin. centers (% of offenses)	2016
Hospital migration to other regions (% of hospitalized inhabitants from region)	2016
Newly registered non-agricultural companies (% of comp. registered in the prev. year)	2018
Net increase in registered companies (% of companies registered in the previous year)	2018
Frequency of accidental interruptions in the electricity service	2017
Energy from renewable sources (% of total)	2010
Value of exportations in sectors with dynamic world demand (% of total exportations)	2018
People in search of work, age 15-24 (% of workforce in the age class)	2018
People in search of work, age 15-24, female (% of workforce in the age-gender class)	2018

People in search of work, age 15-24, male (% of workforce in the age-gender class)	2018
People in search of work, age >15 (% of workforce in the age class)	2018
People in search of work, age >15, female (% of workforce in the age-gender class)	2018
People in search of work, age >15, male (% of workforce in the age-gender class)	2018
Employed people, age 15-64 (% of workforce in the age class)	2018
Employed people, age 15-64, female (% of workforce in the age-gender class)	2018
Employed people, age 15-64, male (% of workforce in the age-gender class)	2018
Employed people, age >54 (% of workforce in the age class)	2018
Employed people, age >54, female (% of workforce in the age-gender class)	2018
Employed people, age >54, male (% of workforce in the age-gender class)	2018
Absolute difference between male and female employment rate, age 15-64	2018
Absolute difference between male and female activity rate, age 15-64	2018
Workforce, age 15-64 (% of population in the age class)	2018
Female workforce, age 15-64 (% of population in the age-gender class)	2018
Male workforce, age 15-64 (% of population in the age-gender class)	2018
Reported thefts (per 1,000 inhabitants)	2017
Reported robberies (per 1,000 inhabitants)	2017
Voluntary manslaughters (per 100,000 inhabitants)	2017
Decay rate of cash credit lines (%)	2018
Patents registered at the European Patent Office (EPO) (per 1M inhabitants)	2012
Separate collection of urban waste (% of total collection)	2017
Collected urban waste (kg)	2017
Landfilled urban waste (kg per inhabitant)	2011
Landfilled urban waste (% of total urban waste)	2011
Supplied water (% of water fed into the urban distribution network)	2009
Elderly people (age >65) treated in socio-assistential home assistance (% of elderly p.)	2015
Municipalities that have activated childhood services (% of municipalities in province)	2016
Children (age 0-3) that benefited from childhood services (% of pop. in the age class)	2015
Ultra-broadband subscriptions (% of inhabitants)	2017
Index of accessibility to urban and logistic nodes	2013
Disembarked and boarded airline passengers (per 100 inhabitants)	2017
Days of stay in accommodation facilities in non-summer months (per inhabitant)	2017
Days of stay in accommodation facilities (per inhabitant)	2017
Welfare and sustainability indicators	
Life expectancy at birth (years)	2017
Life expectancy at birth, male (years)	2017
Life expectancy at birth, female (years)	2017
Child mortality (per 1,000 born alive)	2016
Road accident mortality, age 15-34 years (per 10,000 inhabitants in the class)	2017
Road accident mortality, age 15-34 years, male (per 10,000 inhabitants in the class)	2017
Road accident mortality, age 15-34 years, female (per 10,000 inhabitants in the class)	2017
Cancer mortality, age 20-64 years (per 10,000 inhabitants in the class)	2016
Cancer mortality, age 20-64 years, male (per 10,000 inhabitants in the class)	2016
Cancer mortality, age 20-64 years, female (per 10,000 inhabitants in the class)	2016
Dementia and neurological disease mortality, age >65 (per 10,000 inh. in the class)	2016

Dementia and neurological disease mortality, age >65, male (per 10,000 inh. in class)	2016
Dementia and neurological disease mortality, age >65, female (per 10,000 inh. in class)	2016
Attendance rate in preschool (%)	2017
Population with high-school diploma, age 25-64 (%)	2018
Population with university degree and other superior qualifications, age 25-39 (%)	2018
Transition to university (specific cohort rate)	2018
Young NEET population (%)	2018
Participation to continuing education (%)	2018
Alphabetic competence of students (average score)	2018
Alphabetic competence of students, male (average score)	2018
Alphabetic competence of students, female (average score)	2018
Mathematical competence of students (average score)	2018
Mathematical competence of students, male (average score)	2018
Mathematical competence of students, female (average score)	2018
Occupation rate, age 20-64 (%)	2018
Occupation rate, age 20-64, male (%)	2018
Occupation rate, age 20-64, female (%)	2018
Rate of lack of participation in work (%)	2018
Rate of lack of participation in work, male (%)	2018
Rate of lack of participation in work, female (%)	2018
Rate of work accidents causing death or permanent disability (per 10,000 workers)	2016
Rate of work accidents causing death or p. disability, male (per 10,000 w. in class)	2016
Rate of work accidents causing death or p. disability, female (per 10,000 w. in class)	2016
Youth occupation rate, age 15-29 (%)	2018
Youth occupation rate, age 15-29, male (%)	2018
Youth occupation rate, age 15-29, female (%)	2018
Rate of youth lack of participation in work, age 15-29 (%)	2018
Rate of youth lack of participation in work, age 15-29, male (%)	2018
Rate of youth lack of participation in work, age 15-29, female (%)	2018
Working days per year for employees (%)	2017
Average per capita income (EUR)	2016
Average yearly wage for employees (EUR)	2017
Average yearly wage for employees, male (EUR)	2017
Average yearly wage for employees, female (EUR)	2017
Average yearly amount of retirement incomes (EUR)	2017
Average yearly amount of retirement incomes, male (EUR)	2017
Average yearly amount of retirement incomes, female (EUR)	2017
Retired population with low income (%)	2017
Retired population with low income, male (%)	2017
Retired population with low income, female (%)	2017
Wealth per capita (EUR)	2016
Inflow rate of bad loans to families (%)	2017
Non-profit organizations (per 10,000 inhabitants)	2016
Accessible schools (%)	2018
Voter turnout, European elections (%)	2014

Voter turnout, regional elections (%)	2018
Female municipal administrators (%)	2018
Municipal administrators of age <40 (%)	2018
Penal institution overcrowding (%)	2018
Collection capacity of municipalities (%)	2016
Collection capacity of provinces (%)	2016
Homicides (per 10,000 inhabitants)	2017
Other reported violent crimes (per 10,000 inhabitants)	2017
Reported diffuse offenses (per 10,000 inhabitants)	2017
Road accident mortality on suburban roads (%)	2017
Density and relevance of museum heritage (per 100 km^2)	2017
Diffusion of farmhouse companies (per 100 km^2)	2017
Density of historical green areas (m^2 per 100 m^2 in urban areas)	2017
Dispersion from municipal water supply (%)	2015
Landfilled urban waste (%)	2017
Urban air quality: PM10 (%)	2017
Urban air quality: nitrogen dioxide (%)	2017
Urban green space (m^2 per inhabitant)	2017
Energy from renewable sources (%)	2017
Separate collection of urban waste (%)	2017
Soil sealing with artificial covering (%)	2017
Employees in cultural enterprises (%)	2016
Mobility of Italian graduates, age 25-39 (per 1,000 graduate inhabitants)	2017
Children that benefited from childhood services (%)	2016
Irregularities of electricity service (per inhabitant)	2016
Seats per km offered by local public transport services (per inhabitant)	2016
Hospital migration to other regions (%)	2016
Hospital migration to other regions (%)	2016

Table S6.

Classification according to the International Standard Classification of Education (ICSED) of the Italian degree categories. Information is used for the construction of the Italian educational offer network. Both bachelor (L) and master (LM) degree courses are included (80,81).

**ISCED broad field**	**Italian degree category**
Education	L-19, LM-50, LM-57, LM-85, LM-85 bis, LM-93
Arts and humanities	L-01, L-03, L-04, L-05, L-10, L-11, L-12, L-42, LM-02, LM-12, LM-14, LM-15, LM-36, LM-37, LM-38, LM-39, LM-45, LM-64, LM-65, LM-78, LM-84, LM-89, LM-94, LMR/02
Social sciences, journalism and information	L-06, L-16, L-20, L-24, L-33, L-36, L-37, L-40, L/DS, LM-01, LM-05, LM-19, LM-43, LM-51, LM-52, LM-55, LM-56, LM-59, LM-62, LM-63, LM-80, LM-81, LM-88, LM-90, LM-92, LM/DS
Business, administration and law	L-14, L-18, L/GASTR, LM-16, LM-76, LM-77, LM/GASTR, LM/SC-GIUR, LMG/01
Natural sciences, mathematics and statistics	L-02, L-13, L-27, L-28, L-29, L-30, L-32, L-34, L-35, L-41, L-43, LM-06, LM-07, LM-08, LM-09, LM-11, LM-17, LM-40, LM-54, LM-58, LM-60, LM-61, LM-71, LM-72, LM-74, LM-75, LM-79, LM-82, LM-83
Information and Communication Technologies (ICTs)	L-31, LM-18, LM-66, LM-91
Engineering, manufacturing and construction	L-07, L-08, L-09, L-17, L-21, L-23, LM-03, LM-04, LM-04cu, LM-20, LM-21, LM-22, LM-23, LM-24, LM-25, LM-26, LM-27, LM-28, LM-29, LM-30, LM-31, LM-32, LM-33, LM-34, LM-35, LM-44, LM-48, LM-53
Agriculture, forestry, fisheries and veterinary	L-25, L-26, L-38, LM-42, LM-69, LM-70, LM-73, LM-86
Health and welfare	L-39, L/SNT1, L/SNT2, L/SNT3, L/SNT4, LM-13, LM-41, LM-46, LM-67, LM-87, LM/SNT1, LM/SNT2, LM/SNT3, LM/SNT4
Services	L-15, L-22, LM-47, LM-49, LM-68

4 Default parameters of the Spin Glass community detection algorithm

Community detection is performed using the Spin Glass algorithm (85,86) of the *igraph* Python library (igraph). While the variational parameters are discussed in the main text, we report here the choices made for the default parameters:

- `spins` is the parameter that sets the upper limit for the number of communities. It is fixed to the number of nodes in the network or in the previous-level community that is going to be partitioned, according to the stage of the hierarchical algorithm. An exception is represented by the OECD educational offer network, since the algorithm does not support a number of communities as large as 1088; therefore, only in that case the value of `spins` is set to $1088/4 = 272$ communities.

- `implementation` is a value that can be set to either `default`, if one wants to use the faster original implementation, or to `neg`, if one wants to take into account negative weights; the parameter is set to `neg` (the choice is irrelevant in the case of the educational offer networks).
- `lambda_` is the argument that specifies the balance between the importance of present and missing negatively-weighted edges within a community. Smaller values of lambda lead to communities with less negative intra-connectivity. This value is set to 0.01, and the results are very stable with respect to its variations.
- `update_rule` specifies the null model of the simulation. Possible values are `config`, which sets the model as a random graph with the same vertex degrees as the input graph, and `simple`, in which the model is a random graph with the same number of edges. The value is set to `config`.
- `cool_fact` represents the cooling factor for the simulated annealing process that leads to the optimal configuration. The value is set to 0.5, with the results being very weakly dependent on it.

List of supplementary files

Data S1. (separate file)
Full list of OECD universities with their community membership in both the territorial and educational offer networks.

Data S2. (separate file)
Full list of OECD universities with the debiasing parameters (δ_T, δ_E) and the related principal component values PC1 and PC2, referred to the *THE overall* score and to all its dimensions.

Data S3. (separate file)
Full list of Italian universities with their community membership in both the territorial and educational offer networks.

Data S4. (separate file)
Full list of Italian universities with the debiasing parameters (δ_T, δ_E) and the related principal component values PC1 and PC2, referred to the *CENSIS overall* score and to all its dimensions.

References (from main text)

65. Times Higher Education, World University Rankings. https://www.timeshighereducation.com/content/world-university-rankings. Accessed: 15 July 2021.

67. MapChart - Create your own custom map. https://mapchart.net. Accessed: 1 February 2022.

75. OECD, *OECD Regional Statistics (database)* (OECD, Paris, 2020). http://dx.doi.org/10.1787/region-data-en

78. Misure del Benessere dei territori. https://www.istat.it/it/archivio/230627. Accessed: 15 July 2021.

79. Indicatori territoriali per le politiche di sviluppo. https://www.istat.it/it/archivio/16777/. Accessed: 15 July 2021.

80. MIUR – Organizzazioni – Open Data dell'istruzione superiore. http://dati.ustat.miur.it/organization/miur. Accessed: 15 July 2021.

81. UNESCO Institute for Statistics, *ISCED fields of education and training 2013 (ISCED-F 2013)* (UNESCO Institute for Statistics, Montreal, 2013).

85. J. Reichardt, S. Bornholdt, Statistical mechanics of community detection. *Phys. Rev. E* **74**, 016110 (2006).

86. V.A. Traag, J. Bruggeman, Community detection in networks with positive and negative links. *Phys. Rev. E* **80**, 036115 (2009).

87. S.S. Shapiro, M.B. Wilk, An analysis of variance test for normality (complete samples), *Biometrika* **52**, 591-611 (1965).

88. Ž. Ivezić, A. Connolly, J. Vanderplas, A. Gray, Statistics, *Data Mining and Machine Learning in Astronomy* (Princeton University Press, Princeton, NJ, 2014).
